# Statistical methods and data visualisation of patient-reported outcomes in early phase dose-finding oncology trials: a methodological review

**DOI:** 10.1016/j.eclinm.2023.102228

**Published:** 2023-09-21

**Authors:** Emily Alger, Anna Minchom, Olalekan Lee Aiyegbusi, Matthew Schipper, Christina Yap

**Affiliations:** a Clinical Trial and Statistics Unit, Institute of Cancer Research, London, UK; b Drug Development Unit, Royal Marsden/Institute of Cancer Research, London, UK; c Centre for Patient Reported Outcomes Research, Institute of Applied Health Research, College of Medical and Dental Sciences, University of Birmingham, UK; d National Institute for Health and Care Research (NIHR) Birmingham Biomedical Research Centre, University of Birmingham, Birmingham, UK; e Departments of Radiation Oncology and Biostatistics, University of Michigan, Ann Arbor, MI, USA

**Keywords:** Patient-reported outcomes, Dose-finding, Methodological review, Oncology, Statistical methods

## Abstract

**Background:**

Traditionally, within dose-finding clinical trials, treatment toxicity and tolerability are assessed by clinicians. Research has shown that clinician reporting may have inadequate inter-rater reliability, poor correlation with patient reported outcomes, and under capture the true toxicity burden. The introduction of patient-reported outcomes (PROs), where the patient can assess their own symptomatic adverse events or quality of life, has potential to complement current practice to aid dose optimisation. There are no international recommendations offering guidance for the inclusion of PROs in dose-finding trial design and analysis. Our review aimed to identify and describe current statistical methods and data visualisation techniques employed to analyse and visualise PRO data in published early phase dose-finding oncology trials (DFOTs).

**Methods:**

DFOTs published from June 2016–December 2022, which presented PRO analysis methods, were included in this methodological review. We extracted 35 eligible papers indexed in PubMed. Study characteristics extracted included: PRO objectives, PRO measures, statistical analysis and visualisation techniques, and whether the PRO was involved in interim and final dose selection decisions.

**Findings:**

Most papers (30, 85.7%) did not include clear PRO objectives. 20 (57.1%) papers used inferential statistical techniques to analyse PROs, including survival analysis and mixed-effect models. One trial used PROs to classify a clinicians’ assessed dose-limiting toxicities (DLTs). Three (8.6%) trials used PROs to confirm the tolerability of the recommended dose. 25 trial reports visually presented PRO data within a figure or table within their publication, of which 12 papers presented PRO score longitudinally.

**Interpretation:**

This review highlighted that the statistical methods and reporting of PRO analysis in DFOTs are often poorly described and inconsistent. Many trials had PRO objectives which were not clearly described, making it challenging to evaluate the appropriateness of the statistical techniques used. Drawing conclusions based on DFOTs which are not powered for PROs may be misleading. With no guidance and standardisation of analysis methods for PROs in early phase DFOTs, it is challenging to compare study findings across trials. Therefore, there is a crucial need to establish international guidance to enhance statistical methods and graphical presentation for PRO analysis in the dose-finding setting.

**Funding:**

EA has been supported to undertake this work as part of a PhD studentship from the 10.13039/501100000650Institute of Cancer Research within the 10.13039/501100000265MRC/10.13039/501100000272NIHR Trials Methodology Research Partnership. AM is supported by the 10.13039/501100000272National Institute for Health Research (NIHR) 10.13039/100014461Biomedical Research Centre at the 10.13039/100012139Royal Marsden NHS Foundation Trust, the 10.13039/501100000650Institute of Cancer Research and Imperial College.

**Research in context**

**Evidence before this study**

Previous reviews have revealed that though patient-reported outcome (PRO) endpoints are only present within a limited number of dose-finding oncology trials (DFOTs), its use has increased over time. There are no guidelines on how PROs should be incorporated in DFOTs, analysed and interpreted, and the quality of PRO analysis in this setting is unknown. To evaluate PRO analysis techniques within DFOTs, we searched the bibliographic database MEDLINE (via PubMed) for eligible studies. Oncology trial reports or trial protocols which were published between June 2016 and December 2022 and included PRO analysis methods within the dose-finding component were included.

**Added value of this study**

To the best of our knowledge, this is the first study providing a contemporary review of statistical and graphical PRO analysis techniques in DFOTs. In line with the introduction of CONSORT-PRO, SPIRIT-PRO, and the founding of the SISAQOL consortium, it is becoming increasingly important to encourage quality PRO reporting within trials. We highlight the benefits of incorporating PROs within dose selection decisions as a long-term approach to assess treatment tolerability and identify methodological weaknesses and recommendations with regards to current analysis practice.

**Implications of all the available evidence**

This review highlights the lack of standardisation and consistency of PRO analysis in DFOTs. This work strengthens the call for new PRO analysis recommendations within the early phase dose-finding setting. Looking forward, the development of guidance to analyse and report PROs will facilitate the interpretation of PRO findings at different dose levels and will aid dose adaptation decisions and final dose selection. Development of guidance will not only generate analysis methods which can contribute to more complete treatment tolerability profiles but may also improve the accuracy of dose determination methods and enable a more accurate synthesis of PRO data in order to achieve patient-centred clinical development.

## Introduction

Within early phase dose-finding oncology trials (DFOTs), the safety and tolerability of a new agent is assessed. Often the recommended phase II dose (RP2D) has generally been set at or close to the maximum tolerated dose (MTD). The MTD is often determined by observing dose-limiting toxicities (DLTs) within patients enrolled on a trial. Clinicians usually grade toxicities using the National Cancer Institute Common Terminology Criteria for Adverse Events (NCI-CTCAE), with grade three or above toxicities considered a DLT.^1^

This approach to MTD or RP2D estimation relies solely on a clinician assessment of tolerability and is not informed by a patient’s own evaluation of their quality of life whilst receiving treatment.^2^ Whilst clinicians can assess adverse events (AEs) such as fever and blood profiles, other AEs such as fatigue or nausea can be subjective toxicities which may be difficult for them grade consistently and which they may undercapture.^3^, ^4^, ^5^, ^6^

Furthermore, the traditional DLT assessment window (usually one or two cycles of treatment) may not be efficient for therapies which are administered for extended periods of time. Oncology treatments are often administered until disease progression (treatment resistance) is observed^7^ and therefore, the desired assessment window to assess treatment tolerability may elongate.^1^ Whilst radiotherapy treatments often have longer DLT assessment periods,^8^^,^^9^ the short DLT assessment window typically used to assess the tolerability of cytotoxic agents may not capture the toxicities beyond the first cycle of treatment. A retrospective study by Postel-Vinay et al. found that 57% of grade three or four toxicities experienced by patients treated with a molecularly targeted agent (MTA) during a phase I trial occurred beyond the first cycle of treatment.^7^ Additionally, the current approach to tolerability assessment does not typically capture the toxicity associated with prolonged Grade 2 toxicities. These types of toxicities may become an impediment to treatment tolerability over longer term dosing schedules. For example, Durvalumab was recently approved to be given for 12 months following chemo-radiation in for locally advanced non-small cell lung cancer. However, many patients discontinue early due to treatment toxicity.^10^

There is growing interest in the introduction of Patient-Reported Outcomes (PROs) within early phase dose-finding trials to inform tolerability of treatment. The US Department of Health defines a PRO as “any report of the status of a patient’s health condition that comes directly from the patient, without interpretation of the patient’s response by a clinician or anyone else”.^11^ Incorporating PROs within DFOTs may enhance researchers’ understanding of toxicity profiles and improve the accuracy of RP2D determination.^12^

The U.S. Food and Drug Administration’s (FDA) Project Optimus initiative encourages the leveraging of clinical and non-clinical data to aid dose optimization within pre-marketing drug development.^13^ Against this backdrop, Friends of Cancer Research have encouraged the introduction of PROs to guide drug optimization.^14^ A systematic review by Lai-Kwon et al. found that only 5.3% (548/10,372) of trials presented on ClinicalTrials.gov in 2007–2020 contained a PRO endpoint, though its use has increased significantly over time.^15^ Currently, as PRO endpoints are only present in a small number of DFOTs, there is limited literature encouraging routine practice in their analysis across the whole trial process, not least dose-finding trials.^16^ Inclusion of PROs in DFOTs has the potential to more accurately characterize toxicity and tolerability as they vary with treatment dose.

New guidance for the inclusion of PROs within interventional clinical trials has been developed to ensure comprehensive publishing of PRO data. CONSORT-PRO^17^ (Consolidated Standards of Reporting Trials–PRO) and SPIRIT-PRO^18^ (Standard Protocol Items: Recommendations for Interventional Trials-PRO) were developed to ensure the rigorous reporting of PROs within trial reports and trial protocols. Additional work has been undertaken to introduce guidance for statistical analysis plans (SAPs) in early phase clinical trials,^19^ alongside ongoing development of the SPIRIT and CONSORT extensions for early phase dose-finding trials.^20^^,^^21^ The newly founded SISAQOL Consortium (Setting International Standards in Analyzing Patient-Reported Outcomes and Quality of Life Endpoints Data) have generated recommendations to adapt statistical methods, missing data and statistical terminology for the incorporation of PROs within cancer randomized controlled trials (RCTs).^22^ However, there is currently no international guidance for PRO statistical analysis in early phase DFOTs,^12^^,^^23^ which are typically non-randomized.^24^ It is unclear which PRO statistical analysis strategies are currently being employed within DFOTs.

To assess the current PRO analysis methods and data visualization techniques utilised within dose-finding oncology trials we conducted a methodological review via PubMed.

Our objectives were to: describe the study characteristics of published trials investigating PROs within early phase DFOTs, identify and evaluate current techniques to analyse PRO measures within early phase DFOTs, and to explore the data visualisation techniques used to display PRO measures graphically.

## Methods

### Study strategy and selection criteria

Papers eligible for this methodological review were dose-finding oncology trials or trial protocols archived on PubMed between 01/06/2016 and 31/12/2022 which described how PRO data was to be analysed. For a trial protocol, the paper should include a presentation of the statistical techniques which the researchers planned to use to analyse PRO data. For a trial report, the paper should include a presentation of the statistical techniques which were used to analyse PRO data collected during the study. Clinical trials were extracted by EA in XML format on 21/04/2023.

Eligible papers were extracted using the following search strategy: ("dose-find∗" OR "dose escalat∗" OR "dose find∗" OR "dose expan∗" OR "single ascending dose" OR "multiple ascending dose" OR "first in man" OR "first in human" OR "early phase" OR "phase 1a" OR "phase 1b" OR "phase ia" OR "phase ib" OR "RP2D") AND ("quality of life" OR "patient reported outcome∗" OR "patient-reported outcome∗") AND ("2016/06/01" [Date–Completion]: "2022/12/31" [Date–Completion]).

Each entry was reviewed for eligibility by one reviewer (EA). Papers were eligible if they:

(1) Reported a dose-finding component within an early phase trial with a cancer population,
(2) The intervention of interest was either a drug or radiotherapy,
(3) PRO analysis was presented within the dose-escalation component.

For data verification of eligibility of papers, 9.6% (54/562) of randomly selected papers were double-reviewed (EA and AM) to ensure eligible papers were captured. For data extraction, 14.3% (5/35) of randomly selected eligible papers were assessed by an additional reviewer (AM, OLA, CY) to ensure all relevant features were correctly extracted. All queries that arose during data extraction were discussed and any differences of opinions between reviewers were resolved through discussion.

### Data analysis

For eligible papers, the following characteristics were extracted: year of publication, study population, cancer type, trial phase, anti-cancer agent, funder type, number of centers, trial design, PRO instrument, frequency of PRO assessment, minimal clinically important difference (MCID), primary endpoint. We also extracted additional PRO statistical features including: PRO objectives, PRO analysis method, PRO visualisation technique, discussion of missing PRO data reported, number of patients with missing PRO data, reasons described for missing PRO data, and methodology to manage missing PRO data described.

To evaluate the use of PROs across a diverse range of trial demographics, we firstly extracted basic trial characteristics. Extracted trial characteristics were a subset of those in Yap et al.'s^24^ review on the quality of early phase DFOTs. To evaluate the statistical rigor of PRO methods and analysis, we extracted features which provided a general overview of statistical methodology. These included items extracted by Lai-Kwon et al.^15^ in their ClinicalTrials.gov review of PROs in DFOTs. In addition, we collected PRO objectives, how PRO analysis was attempted, how it was presented to the reader (data visualisation) and how missing data was recorded and mitigated. The option categories for each feature are presented in Tables 1, 2, 3, and 4.

**Table 1:** Characteristics of eligible early phase dose-finding oncology trials.

	Overall (N = 35)
Trial design	
Algorithmic	22 (62.9%)
3 + 3	16 (45.7%)
Rolling six	4 (11.4%)
Other	2 (5.7%)
Model based	7 (20.0%)
Continual reassessment method (CRM)	5 (14.3%)
Time-to-event continual reassessment method (TiTE-CRM)	1 (2.9%)
Escalation with overdose control (EWOC)	1 (2.9%)
Unclear	6 (17.1%)
Intervention type	
Drug	17 (48.6%)
Drug + radiotherapy	5 (14.3%)
Radiotherapy	13 (37.1%)
Number of PRO measures	
1	15 (42.9%)
2	11 (31.4%)
3	8 (22.9%)
5	1 (2.9%)
Number of PRO assessments	
Mean (SD)	6.31 (4.75)
Median [min, max]	5.00 [2.00, 24.0]
Type of PRO analysis	
Descriptive	15 (42.9%)
Descriptive & inferential	11 (31.4%)
Inferential	9 (25.7%)
PRO endpoint	
Exploratory	1 (2.9%)
Secondary	23 (65.7%)
Tertiary	1 (2.9%)
Unclear	10 (28.6%)

A full table presenting a summary of all extracted features is presented in Supplementary Table S2.

**Table 2:** Type and number of each PROM questionnaire captured within this review, along with the associated trials which utilised each questionnaire.

Questionnaire	Number of times used as instrument	Associated trials
EORTC QLQ-C30 (Quality of life of cancer patients)	14	^8^^,^^25^, ^26^, ^27^, ^28^, ^29^, ^30^, ^31^, ^32^, ^33^, ^34^, ^35^, ^36^, ^37^
EQ-5D (Generic quality of life)	5	^28^^,^^38^, ^39^, ^40^, ^41^
EPIC	4	42, 43, 44, 45
IPSS	3	^42^^,^^44^^,^^46^
AUA	3	^43^^,^^44^^,^^47^
M.D. Anderson Dysphagia Inventory	2	^8^^,^^28^
CLAS1	2	^48^^,^^49^
CLAS2	2	^48^^,^^49^
CLAS3	2	^48^^,^^49^
FACT-G (General)	2	^50^^,^^51^
EORTC QLQ-H&N35 (Head & neck)	2	^8^^,^^28^
FACT-P (Prostate)	1	^39^
FACT-ES (Endocrine symptoms)	1	^52^
Impact of Pediatric Illness (IPI) Parent Report Form	1	^53^
Norfolk QOL-NET (Neuroendocrine tumour)	1	^54^
FACT-Ga (Gastric)	1	^55^
EORTC QLQ-LC13 (Lung)	1	^25^
VASB	1	^27^
WOMAC	1	^40^
PedsQL	1	^56^
SHIM	1	^46^
EORTC QLQ—STO22 (Gastric)	1	^55^
FACT-KSI (Kidney)	1	^50^
EORTC QLQ-BR23 (Breast)	1	^31^
DLIQ	1	^25^
Rectal function study questionnaire^57^	1	^46^
VHI	1	^28^
EORTC QLQ-PAN26 (Pancreatic cancer)	1	^58^
O’Leary Interstitial Cystitis Symptom Index	1	^47^
EORTC QLQ-BN20 (Brain)	1	^34^
EORTC QLQ-PR25 (Prostate)	1	^35^
FAACT	1	^58^
FACT-BP (Bone pain)	1	^51^
MDASI-BT (Brain tumour)	1	^59^

A table of acronyms used in this table are explained in Supplementary Table 3.

**Table 3:** Description of PRO statistical inferential techniques captured, with associated trials separated by the treatment under investigation.

Statistical inferential technique	Description	Drug trial (n = 8)	Radiotherapy trial (n = 12)
Wilcoxon signed-rank test	Change in PRO between baseline and another timepoint.	^30^	^28^^,^^50^^,^^56^
Wilcoxon rank-sum test	Association between PRO and dosimetric parameters at timepoints.		^28^
Mixed-effect model	Fit model predicting PRO score across study from baseline accounting for intra-patient correlation.	^40^	^8^^,^^51^
	Compare change in PRO score across each dose cohort when time is fitted as a random effect		^43^
Linear Regression	Fit model predicting average PRO score for specific symptoms across study from baseline.	^54^	
t-tests	Change in PRO score between baseline and another timepoint or difference in PRO score between dose cohorts.	^25^^,^^34^^,^^35^^,^^58^	^29^^,^^38^^,^^41^^,^^46^^,^^48^
Fisher's exact test	Association between MCID and each dose cohort		^45^
Survival Analysis	Fit model predicting time from inclusion in study until deterioration or grade 3/4 toxicity using a Cox proportional hazards model.	^33^	

PRO: patient-reported outcome; MCID: minimal clinically important difference; HRQoL: health related quality of life.

**Table 4:** The number and proportion of times each figure type was used to visualise PRO data, with associated trials.

Figure type	Overall (N = 18) (%)	Associated trials
Longitudinal plot	12 (66.7)	^30^^,^^34^^,^^37^^,^^42^, ^43^, ^44^, ^45^, ^46^^,^^51^^,^^52^^,^^54^^,^^59^
Barplot	3 (16.7)	^26^^,^^36^^,^^40^
Box plot	1 (5.6)	^40^
Histogram	1 (5.6)	^41^
Kaplan–Meier graph	1 (5.6)	^33^

### Statistics

For each extracted feature, the percentage of papers which reported each possible outcome was presented. For features with a numerical domain, the corresponding 95% confidence interval (CI) was also calculated. No data imputation was performed.

A linear regression model with year as the independent variable was fitted to assess the trend in the analysis of PROs within DFOTs over time, and model diagnostics were evaluated to assure the suitability of this model. R version 4.2.1 was used for the statistical analysis.

### Role of funding source

EA has been supported to undertake this work as part of a PhD studentship from the Institute of Cancer Research within the MRC/NIHR Trials Methodology Research Partnership.

AM is supported by the National Institute for Health Research (NIHR) Biomedical Research Centre at the Royal Marsden NHS Foundation Trust, the Institute of Cancer Research and Imperial College. The views expressed are those of the authors and not necessarily those of the NIHR or the Department of Health and Social Care.

No funders had a role in the data collection, data analyses, interpretation, or writing of this report.

## Results

**Fig. 1:**
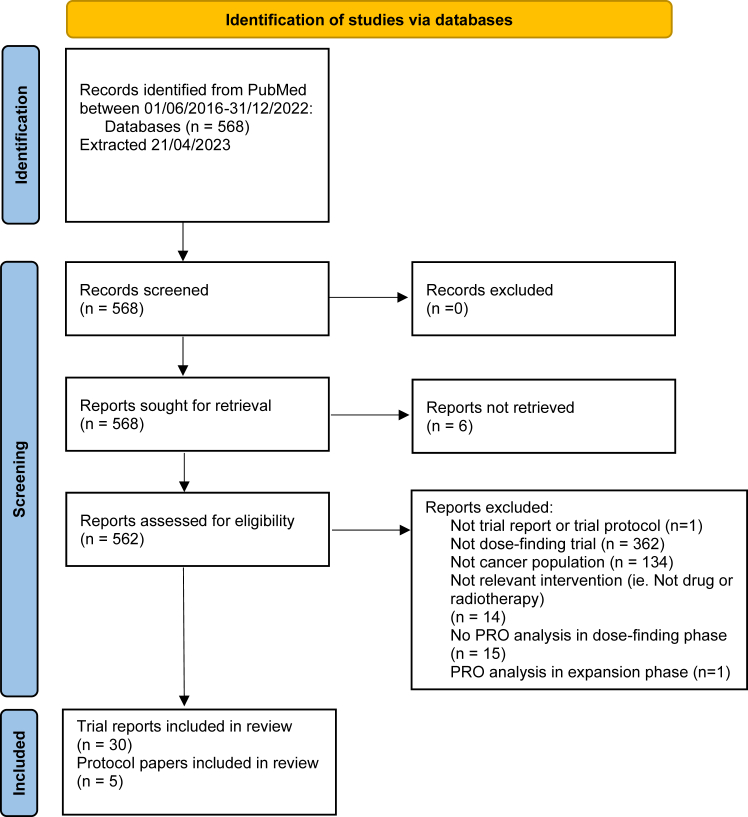
PRISMA study flow diagram illustrating selection of eligible studies.^60^

562 papers were assessed for eligibility and 35 papers were eligible for the review. A study flow diagram for this study is presented in Fig. 1. There was 94.4% agreement between reviewers (EA and AM) assessing eligible trials. For the three papers where EA and AM disagreed on paper eligibility, disagreements were resolved by discussion with additional arbitrators (OLA and CY).

30 of the eligible manuscripts detailed completed trials. Five manuscripts were published trial protocols.^8^^,^^38^^,^^39^^,^^47^^,^^55^ Supplementary Table S1 summarises the eligible papers analysed within this methodological review.

An overview of papers included in this review is presented in Table 1. When PROs were labelled as an endpoint (n = 25), it was most often identified as a secondary endpoint (23, 92.0%). Two papers marked the PRO outcome as an exploratory or tertiary endpoint. All papers which presented a PRO endpoint also presented the PRO analysis within the paper.

34 distinct questionnaires were used to evaluate PROs and are presented in Table 2. Often papers utilized more than one patient-reported outcome measure (PROM). Each paper considered a median of 2 questionnaires (Range: 1–4).

Notably, no eligible trials considered the NCI-PRO-CTCAE questionnaire. This criterion was developed in 2014 by the National Cancer Institute to complement the NCI-CTCAE criteria used by clinicians to assess treatment toxicities.^61^

The majority of eligible papers which stated their trial design (29, 82.9%) used a 3 + 3 dose escalation design (16, 45.7%), however Rolling six, (TiTE)-CRM, EWOC, and other specific algorithmic designs were also present within the review. Nearly all (33, 94.3%) trials considered an adult population, however two considered pediatric populations.^53^^,^^56^ In these cases, pediatric specific quality of life questionnaires were considered (PedsQL and Impact of Pediatric Illness (IPI) Parent Report Form).

Patient-reported outcomes were only considered as part of the dose-finding decisions in four (11.4%) trials.^30^^,^^32^^,^^33^^,^^47^ In three cases, the maximum tolerated dosages were confirmed using the usual dose escalation design (CRM (2) and 3 + 3 (1)) and the PROs were used to confirm the tolerability of the recommended phase 2 dose (RP2D). PROs were used to determine whether the MTD was tolerable from a quality of life perspective after the MTD had been determined. In one case, a specific rise in PRO score (signaling a deterioration in health related quality of life) was defined as a DLT and used to guide 3 + 3 dose escalation.^47^

There was no significant time trend in the number of published trials which reported PRO analysis (0.25, 95% CI: −1.46 to 1.96). The mean number of time points PROs were assessed for a drug intervention was 4.88 (SD: 3.12). The mean number of time points PROs were assessed for a radiotherapy, or drug and radiotherapy combination trial was 7.67 (SD: 5.66).

### Analysis strategies

18 (51.4%) extracted manuscripts provided information on the planned analysis of PROs in the methods section of the paper. In the majority of papers (85.7%), the PRO objective was not explicitly stated or it was vague, for example “assessing quality of life”. Five papers defined a minimally important clinical difference (MCID) for patients’ quality of life deterioration. 15 (42.9%) papers considered only exploratory statistical analysis, these methods included: plotting quality of life scores over time for each patient, and considering average, median and IQR scores for each question. The majority of papers (n = 20) considered inferential, or explanatory and inferential statistics in their analysis. 14 papers (40.0%) used hypothesis tests to analyse PROs, no papers mentioned whether this test was powered. In general, these hypothesis tests were used to assess whether there was a statistically significant difference in PRO scores across time points or across dose cohorts. Only one paper checked model assumptions before model fitting. Anota and colleagues^33^ tested the proportional hazard assumption using Schoenfeld residuals before utilising a cox proportional hazard model to predict time to quality of life deterioration. No papers completed any form of model validation. Of the 20 papers which utilised some inferential statistics, 13 papers reported the statistical software used. Software included SAS (n = 5), R (n = 4), SPSS (n = 2), Stata (n = 2).

Of the 30 trial reports eligible for this review, only six papers commented on the number of patients with missing data at PRO assessments. The median number of patients who had at least one missing PRO assessment was 1 (range: 0–2). No paper presented the reason for the missing PRO data.

An overview of the statistical techniques extracted within this review is presented in Table 3.

### Presentation of PRO results using figures or tables

80% of trial reports (n = 24/30) visually presented PRO data within a figure or table within their publication. Nine trial papers (30.0%) presented PRO data within a table and 17 trial papers (56.7%) presented PRO data within a figure. 11 papers (36.7%) presented PRO results over each dosage within either a figure or table.

Within the 17 papers which included figures presenting PRO data, 18 distinct plots were identified. The number of times each figure type was used to visualise PRO data is presented in Table 4.

**Fig. 2:**
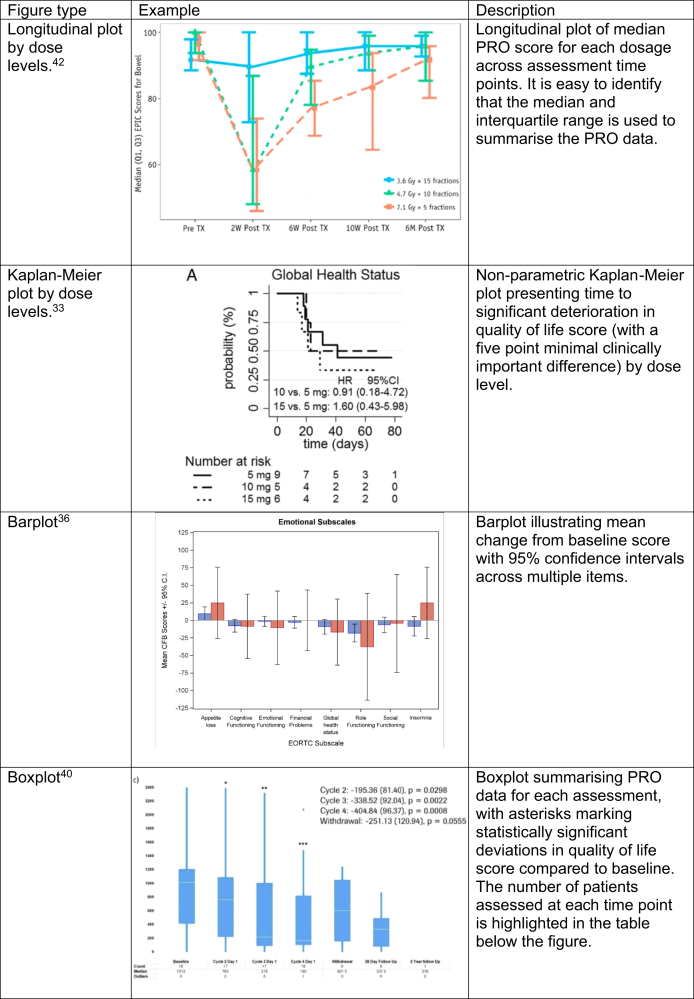
Exemplar plots visualising PRO data identified within this review.

**Fig. 3:**
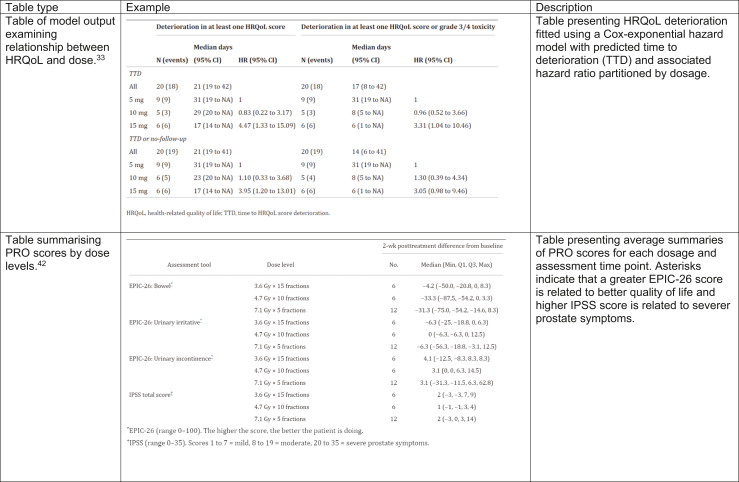
Exemplar tables visualising PRO data identified within this review.

PRO data visualisation methods extracted during this review are presented in Figs. 2 and 3.

Out of the 18 figures, 7 (38.9%) presented PRO data by dose levels, 6 of these figures were longitudinal plots and 1 was a Kaplan–Meier plot. 12 figures plotted PRO scores longitudinally across PRO assessment timepoints. Of these 12 plots, five (41.7%) used a larger score to signify an improved quality of life whereas seven (58.3%) used a larger score to signal a worse quality of life. One paper plotted each individual’s overall score.^52^ Nine figures presented either the mean or median PRO score for each dose cohort. Two papers plotted some PRO statistic longitudinally but did not label whether the mean or median score was used. Four papers used barplots, boxplots, or histograms to visualise PRO data. One paper presented the proportion of patients which experienced an improvement or deterioration in quality of life between their baseline and last PRO assessment,^26^ and one paper used Kaplan–Meier graphs to show the deterioration of quality of life across dose, this plot is presented in Fig. 2.^33^ One paper presented box plots of PRO score for each cycle of treatment (see Fig. 2) and a barplot to show the proportion of patients who experienced an improvement or worsening of symptoms at each cycle of treatment. In this paper, asterisks were used above the boxplots to signify statistically significant changes in quality of life from baseline.^40^ This was mirrored by another paper which presented a barplot of mean scores for different symptoms for the determined MTD and other dosages (see Fig. 2).^36^ One paper used a histogram to show the distribution of PRO scores.^41^

Of the nine tables, eight presented descriptive summaries of the PRO scores and one table presented fitted model results. Two papers presented PRO scores as means and standard deviations: one determined the average score before and after treatment^25^ and one determined the average difference in score compared to baseline.^32^ Four papers presented median PRO scores alongside inter-quartile ranges or ranges. Of these papers, one summarised the PROs by each item and three summarised PROs by each questionnaire used to assess quality of life. One other table highlighted the proportion of each cohort which experienced every symptom before and after treatment. Of the nine tables, four (44.4%) presented PRO data at every time point and not at each dose level. The remaining five tables (55.5%) recorded PROs for each dosage and timepoint separately. One paper presented the median predicted time to deterioration of at least one PRO score and associated hazard score in a table, this is presented in Fig. 3.^33^ Three papers presented p-values associated with un-powered hypothesis tests which hypothesized that quality of life altered from baseline.

## Discussion

Following the recent MDICT 2022 report,^1^ it is increasingly important for trials to identify optimal treatment dosages compared to MTDs in DFOTs. Establishing patient’s views on drug tolerability using Patient-reported outcomes (PROs) may help to assess the effect of dosages on treatment tolerability and quality of life in DFOTs. Only one paper used PRO data to guide dose escalation/de-escalation and three (8.6%) papers made some attempt to use PRO analysis to inform their understanding of drug tolerability. Whilst there remains no specific guidance on the reporting of PROs within early phase dose-finding trials, the SISAQOL guidance and CONSORT-PRO extension should provide some direction for authors publishing PRO analysis within early phase dose-finding trials.^17^ Though CONSORT-PRO offers guidance within the randomised trial setting, many guidelines are conveyable to the dose-finding trial and reflect current deficiencies. For example, Item 22 of CONSORT-PRO recommends that “Patient reported outcome data should be interpreted in relation to clinical outcomes including survival data, where relevant”.^17^

As there is an absence of established standards for PROs within DFOTs, the evaluation criteria used to evaluate papers within this methodological review has been broadly determined by previous reviews. Thus, the features we have extracted build upon the selection of statistical issues which have previously been identified as critical for the analysis of PROs within DFOTs. These criteria remain broadly in line with established guidelines for PRO analysis in RCTs^17^^,^^18^^,^^22^ and early phase SAPs^19^ and ongoing efforts on the development of the SPIRIT and CONSORT extensions for DFOTs.^20^^,^^21^

Exploration of PRO analysis techniques has previously been researched in the Phase 2 setting. Among others, analysis techniques such as generalised estimating equations and ordinal log-linear models were suggested, both of which were not utilised within this review.^62^

34 distinct PROMs were used to evaluate quality of life within this review. It is significant to note that some PROMs identified within this review appear to be non-validated PRO instruments. A radiotherapy trial conducted by Sampath and colleagues^46^ used a rectal function scale^57^ which combined two quality of life assessments–the Prostate Brachytherapy Research Group Protocol PBRG-1 and Radiation Therapy Oncology Group Protocol. Unvalidated PRO measures may not accurately indicate significant changes to quality of life within a trial if items detailing potential patient experiences on treatment are not identified during a rigorous developmental process.

16 papers utilised the EORTC (European Organisation For Research And Treatment Of Cancer) item library as a PROM within this review. Item banks such as this can be used alongside algorithms such as computerized adaptive testing (CAT) to generate short, concise measures capable of evaluating patient symptoms and overall quality of life.^63^ However, utilising such a bank within early phase trials (where relevant items may not be known prior to the commencement of the trial) may mean that relevant adverse events are not reported by a PROM. Conversely, whilst issuing a complete item library within a trial may record an exhaustive list of symptoms experienced by a patient, this questionnaire may be time consuming, burdensome for patients, and infeasible to administer frequently within a trial. Crucial research is required to establish core set of items from PRO item libraries (which could be from EORTC, FACIT (Functional Assessment of Chronic Illness Therapy), or PRO-CTCAE) that adequately capture common and clinically significant treatment-related symptoms for employment in the early phase dose-finding setting. The PRO core set of items must be appropriate to patient groups and trial design/drug types.

For investigators who wish to evaluate an overall burden of side effects within an early phase dose-finding trial, many item libraries contain single questions which evaluate the overall impact of symptomatic toxicities on a patient. This includes the FACT-G item GP5 “I am bothered by side effects of treatment”, part of the FACIT item library. The US Food and Drug Administration (FDA) and Critical Path Institute’s PRO Consortium explored the strengths of such items at a public workshop in 2017.^64^ Single items such as FACT-GP5 were recognised for their simplicity and for supporting patients to weight their side effects. Such measures were encouraged to enrich a therapy’s side effect profile, particularly the consequences of adverse events on a patient’s quality of life.^64^

The diversity of questionnaires used to evaluate PROs may provide some explanation as to why PRO analysis techniques were varied within this review. When PRO objectives were not stated explicitly, it was unfeasible to evaluate whether the statistical analysis approach utilised was appropriate.^65^ All 14 papers which analysed PROs using a hypothesis test did not mention if the test was powered. There is a high risk that these tests were underpowered to undertake formal hypothesis testing due to small sample sizes in typical DFOTs. Just over half of papers which did present PRO analysis considered inferential statistical analysis. Of the three methods which considered some form of model fitting, only one paper presented this model fitting visually.^54^ All inferential statistical methods which were presented in this review were evaluated by SISAQOL. These methods were assessed for the essential/highly desirable statistical attributes agreed by the SISAQOL consortium.^22^ Anota et al.’s trial report is an exemplar paper which presents most features which were desired within this review.^33^ Of note, this paper presents a specific PRO objective, clear PRO endpoint, and utilises an appropriate analysis measure (fitting HRQoL deterioration to a cox proportional hazard model by dose levels) which is reviewed favorably by SISAQOL. The cox proportional hazard model can handle censored data and has within-group statistical relevance when it comes to estimating time to quality of life score deterioration.

Within DFOTs, patients can be withdrawn from a study due to toxicities, progression, or death. It is very likely that studies which collect PROs may have missing data and therefore it is important that missing data is reported and methods which manage missing PRO data are described. However, within this review, only six papers comment on the number of patients with missing data.

A review of the data visualisation techniques currently utilised within the early phase setting suggests that when PRO data was published as a figure or table, a longitudinal plot was most popular. However, barplots were also used to show changes in PRO scores over time. There was heterogeneity in the way PRO scores were presented, with only 36.7% of all trial reports presenting PRO data at each dose level using either a figure or table which limit their usage to inform patients’ perspective of treatment tolerability across doses. The majority of papers related a larger PRO score to a worse quality of life. Within SISAQOL’s preliminary findings, which aim to standardise the graphical visualization of PRO data, the consortium recommends a larger score to indicate better quality of life. They also recommend that intervals are displayed to indicate thresholds to define “improvement” and “deterioration” in quality of life.^22^ The heterogeneity in PRO data visualisation was also discussed by a Consensus Panel of Oncologists, PRO researchers and patients organised by Snyder et al.^66^ This panel recommended greater PRO scores to signify better quality of life, however it was noted that PROM measures should not be changed to conform to this recommendation. Instead, descriptive labels could be used to confirm the interpretation of larger scores with words such as “None”, “Mild”-“Severe”. This panel also recommended the use of line plots to summarise PRO scores to ensure consistent comparison between trial publications. Other publications have reported that clinicians and PRO researchers may misinterpret PRO results due to the variety of PRO measures and how each measure quantified good or poor quality of life.^67^ Research presented by Brundage et al. recommended that different data visualisation techniques be used to present PRO data to patients and clinicians.^68^ This paper suggested that simple linear plots be used to explain PRO score to patients, and recommended the use of normed scores and p-values to tailor PRO data visualisation techniques to a clinician audience.

This methodological review provides the most contemporary picture of PRO usage within early phase DFOTs to date; however it has some limitations. Whilst the search strategy for this review was rigorous, it may be possible that some papers were missed as we did not include specific PROMs within the search criteria. We instead searched for more general terms such as “quality of life” and “patient-reported outcomes”. Nevertheless, this evaluation encompassed a total of 34 unique PRO questionnaires, making it a comprehensive assessment. It may also be the case that more published papers may have been captured if we expanded the intervention beyond a drug or radiotherapy. We acknowledge that conducting an independent validation on 14.3% of the extracted data may not completely eliminate subjectivity. However, we are content that continuous discussion among the authors minimized its impact. Lai-Kwon and colleagues’ review^15^ has previously highlighted that the number of publications which consider a PRO endpoint is increasing over time. The long-term nature of a clinical trial, from trial registration to publication, could mean that DFOTs which are currently analysing PROs may not yet be published and available for this review.

Within this review, no papers consider the PRO-CTCAE questionnaire to evaluate quality of life. This finding is consistent with a systematic review conducted by Fiteni et al., which found that none of the 15 published phase I trials with PRO endpoints from January 2012 to May 2016 utilised PRO-CTCAE.^2^ Interestingly, 2.7% (10/119) of eligible DFOTs used the PRO-CTCAE questionnaire within Lai-Kwon et al.’s^15^ ClinicalTrials.gov review from January 2007 to January 2020. Reasons why we have not found the PRO-CTCAE questionnaire being utilised in this review could include: the long lag time between trial completion and publication of trial results, the likelihood that many early phase dose-finding trials might have remained unpublished,^69^^,^^70^ or that PRO analytical approaches or results within the dose-escalation component of the trials were not reported and hence ineligible. Other reasons could include the associated publication bias for positive trials. Even if PRO-CTCAE data was collected in a trial, a lack of promising results following PRO analysis, or incomplete PRO data might have discouraged authors from including such PRO data in their manuscript. What’s more, clinicians who do not distinguish between symptomatic and quality of life based PROs may be ill-prepared to deploy the NCI-PRO-CTCAE measure, specifically designed for patients to solely evaluate their symptoms. This may also explain why the PRO-CTCAE PROM was not evaluated in this methodological review.

Within this review we made no distinction between PROs assessing health related quality of life and symptomatic adverse events. Due to conceptual differences in assessing symptom severity/toxicities compared to health-related quality of life deterioration, the statistical analysis methods and data visualisation techniques presented in Table 3, Fig. 2, and Fig. 3 may have different rationales and methodological focus depending on the type of PRO being analysed. It may be the case that, for some eligible papers in this methodological review, investigators have used symptomatic and quality of life based PROs synonymously. Studies included within this review have considered quality of life PROMS exclusively, symptomatic PROMs exclusively, or a mixture of quality of life and symptomatic measures. For example, the EORTC questionnaires extracted in this review primarily focuses on health related quality of life, whilst other measures such as the AUA and WOMAC assess treatment symptoms.

In conclusion, currently, a minority of trials analyse PROs within dose-finding oncology trials. There is vast heterogeneity in the way PROs are analysed and subsequently presented within publications, this prevents comparison across study findings. Urgent improvement is needed. Increasing the inclusion of PROs in dose finding trials in a statistically rigorous and consistent manner has the potential to provide meaningful and reliable conclusions of treatment tolerability and improve selection of dose levels that will be evaluated in subsequent trials. This methodological review encourages the introduction of PRO analysis guidelines for dose-finding clinical trials. We recommend further stakeholder engagement is undertaken to ensure consensus driven recommendations for PRO analysis and visualisation. This research can build on the work of the SISAQOL consortium and Snyder et al.’s panel.^66^ Future work needs to ensure that rigorous methods are in place to integrate patients’ experience and perspectives into trial design and guide optimal analysis of PROs. This will help inform treatment tolerability profiles and dose-selection decisions more efficiently and with less arbitrariness, ultimately leading to patient-centered clinical development.

## Contributors

Emily Alger: Conceptualization, Investigation, Formal Analysis, Methodology, Data Curation, Writing–Original Draft, Writing–Review & Editing, Visualization, Project administration. Anna Minchom: Methodology, Validation, Writing–Review & Editing, Supervision. Olalekan Lee Aiyegbusi: Methodology, Validation, Writing–Review & Editing, Supervision. Matthew Schipper: Writing–Review & Editing, Christina Yap: Conceptualization, Investigation, Methodology, Validation, Writing–Review & Editing, Visualisation, Supervision, Project administration, Funding acquisition.

All authors have read and approved the final version of this manuscript. Authors EA, AM, OLA, and CY have verified the underlying data.

## Data sharing statement

The datasets generated during and/or analysed during the current study are available from the corresponding author on reasonable request.

## Declaration of interests

OLA receives funding from the NIHR Birmingham Biomedical Research Centre (BRC), NIHR Applied Research Collaboration (ARC), West Midlands, NIHR Blood and Transplant Research Unit (BTRU) in Precision Transplant and Cellular Therapeutics at the University of Birmingham and University Hospitals Birmingham NHS Foundation, Innovate UK (part of UK Research and Innovation), Gilead Sciences Ltd., Merck, Anthony Nolan, and Sarcoma UK. He declares personal fees from Gilead Sciences, Merck and GlaxoSmithKline outside the submitted work.

AM is funded by the National Institute for Health Research (NIHR) Biomedical Research Centre at the Royal Marsden NHS Foundation Trust. She has served on advisory boards for Janssen Pharmaceuticals, Merck Pharmaceuticals, Takeda Pharmaceuticals and Genmab Pharmaceuticals. Has received honoraria from Chugai Pharmaceuticals, Novartis Oncology, Faron Pharmaceuticals, Bayer Pharmaceuticals, Merck Pharmaceuticals, GSK and Janssen Pharmaceuticals. Has received expenses from Amgen Pharmaceuticals and LOXO Oncology. Has received research funding from Merck Pharmaceuticals and MSD.
