# Supplementary material for: Statistical methods and data visualisation of patient-reported outcomes in early phase dose-finding oncology trials: a methodological review
Source: eClinicalMedicine. 2023 Sep 21;64:102228. doi: 10.1016/j.eclinm.2023.102228 (PMC10541462; doi:10.1016/j.eclinm.2023.102228)
Supplement: Supplementary Material [file mmc1.pdf]

Supplementary Material

Supplementary Table 1.. 2
Supplementary Table 2.. 4
Supplementary Table 3.. 7

Supplementary Table 1

Publication	Year	Trial manuscript type	Study population (Age)	Drug type	PROMs utilised
Goody et al.[30]	2016	Report	Adult	Chemotherapy	EORTC QLQ-C30
Anota et al.[33]	2016	Report	Adult	Chemotherapy	EORTC QLQ-C30
Subbiah et al.[26]	2017	Report	Adult	Chemotherapy	EORTC QLQ-C30
Veldhuijzen van Zanten et al.[56]	2017	Report	Paediatric	Chemotherapy + radiotherapy	PedsQL
Reiss et al.[29]	2017	Report	Adult	Targeted drug (small molecule) + radiotherapy	EORTC QLQ-C30
Heiss et al.[53]	2018	Report	Paediatric	Targeted drug (large molecule)	Impact of Pediatric Illness (IPI) Parent Report Form
Kim et al.[54]	2018	Report	Adult	Targeted drug (small molecule)	Norfolk QOL-NET
Farina et al.[49]	2018	Report	Adult	Radiotherapy	CLAS1, CLAS2, CLAS3
Correa et al.[50]	2018	Report	Adult	Radiotherapy	FACT-G, FACT-KSI
Guiu et al.[32]	2018	Report	Adult	Chemotherapy	EORTC QLQ-C30
Stega et al.[25]	2019	Report	Adult	Amino acid	EORTC QLQ-C30, EORTC QLQ-LC13, DLIQ
Sher et al.[28]	2019	Report	Adult	Radiotherapy	EORTC QLQ-C30, EORTC QLQ-H&N35, M.D. Anderson Dysphagia Inventory, VHI, EQ-5D
Ballas et al.[42]	2019	Report	Adult	Radiotherapy	EPIC, IPSS
Potters et al.[43]	2019	Report	Adult	Radiotherapy	EPIC, AUA
Vatandoust et al.[55]	2019	Protocol	Adult	Chemotherapy + radiotherapy	FACT-Ga, EORTC QLQ–STO22
Diehl et al.[38]	2019	Protocol	Adult	Radiotherapy	EQ-5D
Ippolito et al.[31]	2019	Report	Adult	Radiotherapy	EORTC QLQ-C30, EORTC QLQ-BR23
Alayed et al.[45]	2019	Report	Adult	Radiotherapy	EPIC
Zamagni et al.[48]	2019	Report	Adult	Radiotherapy	CLAS1, CLAS2, CLAS3
Cassier et al.[40]	2020	Report	Adult	Immunotherapy	EQ-5D, WOMAC
Den et al.[44]	2020	Report	Adult	Radiotherapy	EPIC, IPSS, AUA
Kong et al.[8]	2020	Protocol	Adult	Targeted drug (small molecule) + chemotherapy + radiotherapy	EORTC QLQ-C30, EORTC QLQ-H&N35, M.D. Anderson Dysphagia Inventory
Xiao et al.[39]	2020	Protocol	Adult	Hormone therapy + radiotherapy	FACT-P, EQ-5D
Sampath et al.[46]	2020	Report	Adult	Radiotherapy	IPSS, SHIM, rectal function scale

Smith et al.[35]	2020	Report	Adult	Hormone therapy	EORTC QLQ-C30, EORTC QLQ-PR25
Chawla et al.[36]	2020	Report	Adult	Chemotherapy	EORTC QOL-C30
Cohen et al.[59]	2020	Report	Adult	Antibiotic	MDASI-BT
Muller et al.[51]	2020	Report	Adult	Radiotherapy	FACT-G, FACT-BP
Rahman et al.[41]	2020	Report	Adult	Targeted therapy	EQ-5D
Schmidt et al.[52]	2021	Report	Adult	Hormone therapy	FACT-ES
Hocking et al.[27]	2021	Report	Adult	Dietary supplement	EORTC QLQ-C30, VASB
Moe et al.[47]	2021	Protocol	Adult	Immunotherapy	O'Leary Interstitial Cystitis Symptom Index, AUA
Mercier et al.[37]	2021	Report	Adult	Radiotherapy	EORTC QLQ-C30
O'Rawe et al.[34]	2022	Report	Adult	Targeted drug (small molecule)	EORTC QLQ-C30, EORTC QLQ-BN20
Gong et al.[58]	2022	Report	Adult	Immunotherapy + chemotherapy	EORTC QLQ-PAN26, FAACT

Supplementary Table 2

	**Overall (N=35)**
**Study population**	
Adult	33 (94·3%)
Paediatric	2 (5·7%)
**Study phase**	
Phase 1 dose escalation	29 (82·9%)
Phase 1/2	6 (17·1%)
**Funder**	
Government	3 (8·6%)
Industry	6 (17·1%)
Internal funding	8 (22·9%)
Private not-for-profit	9 (25·7%)
Unclear	9 (25·7%)
**Number of centres**	
Multi-centre	7 (20·0%)
Single-centre	21 (60·0%)
Unclear	7 (20·0%)
**Trial design**	
Algorithmic	22 (62·9%)
3+3	16 (45·7%)
Rolling six	4 (11·4%)
Other	2 (5·7%)
Model based	7 (20·0%)
Continual Reassessment Method (CRM)	5 (14·3%)
Time-to-Event Continual Reassessment Method (TiTE-CRM)	1 (2·9%)
Escalation with Overdose Control (EWOC)	1 (2·9%)
Unclear	6 (17·1%)
**Intervention type**	
Drug	17 (48·6%)
Drug + radiotherapy	5 (14·3%)
Radiotherapy	13 (37·1%)
**Drug type**	

Amino acid	1 (2·9%)
Antibiotic	1 (2·9%)
Chemotherapy	5 (14·3%)
Chemotherapy + radiotherapy	2 (5·7%)
Dietary supplement	1 (2·9%)
Hormone therapy	2 (5·7%)
Hormone therapy + radiotherapy	1 (2·9%)
Immunotherapy	2 (5·7%)
Immunotherapy + chemotherapy	1 (2·9%)
Radiotherapy	13 (37·1%)
Targeted drug (large molecule)	1 (2·9%)
Targeted drug (small molecule)	3 (8·6%)
Targeted drug (small molecule) + chemotherapy + radiotherapy	1 (2·9%)
Targeted drug (small molecule) + radiotherapy	1 (2·9%)
**Number of PRO measures**	
1	15 (42·9%)
2	11 (31·4%)
3	8 (22·9%)
5	1 (2·9%)
**Number of PRO assessments**	
Mean (SD)	6.31 (4·75)
Median [Min, Max]	5·00 [2·00, 24·0]
**Type of PRO analysis**	
Descriptive	15 (42·9%)
Descriptive & Inferential	11 (31·4%)
Inferential	9 (25·7%)
**PRO endpoint**	
Exploratory	1 (2·9%)
Secondary	23 (65·7%)
Tertiary	1 (2·9%)
Unclear	10 (28·6%)
**PRO confirms tolerability of RP2D**	
No	31 (88·6%)
Yes	4 (11·4%)
**Primary endpoint**	

Adverse events	1 (2·9%)
MTD	14 (40·0%)
MTD, Feasibility	1 (2·9%)
MTD, RP2D	1 (2·9%)
MTD, RP2D, Tolerability	1 (2·9%)
PSA (Prostate-Specific Antigen) response	1 (2·9%)
RP2D	2 (5·7%)
RP2D, Toxicity	1 (2·9%)
Safety	3 (8·6%)
Safety, Tolerability	5 (14·3%)
Safety, Toxicity	1 (2·9%)
Toxicity	1 (2·9%)
Unclear	3 (8·6%)

Supplementary Table 3

**Abbreviation**	**Definition**
(TiTE)-CRM	(Time to event)-Continual reassessment method
AE	Adverse event
AUA	American Urological Association score
CLAS	Cancer Linear Analog Scales
CONSORT	Consolidated Standards of Reporting Trials
DFOT	Dose-finding oncology trial
DLIQ	Dermatology Life Quality Index
DLT	Dose-limiting toxicity
EORTC	European Organisation For Research And Treatment Of Cancer
EPIC	Expanded Prostate Cancer Index Composite
EQ	EuroQoL
EWOC	Escalation with Overdose Control
FAACT	Functional Assessment of Anorexia-Cachexia Therapy
FACIT	Functional Assessment of Chronic Illness Therapy
FACT	Functional Assessment of Cancer Therapy
FDA	US Food and Drug Administration
HRQoL	Health-related quality of life
IPSS	International Prostate Symptom Score
MCID	Minimal clinically important difference
MDASI	MD Anderson Symptom Inventory
MDICT	Methodology for the Development of Innovative Cancer Therapies
MTD	Maximum tolerated dose
NCI-CTCAE	National Cancer Institute – Common Terminology Criteria for Adverse Events
PRO	Patient-reported outcome
PROM	Patient-reported outcome measure
PSA	Prostate-Specific Antigen
RCT	Randomised control trial
RP2D	Recommended phase 2 dose
SAP	Statistical analysis plan
SHIM	Sexual Health Inventory for Men
SISAQOL	Setting International Standards in Analysing Patient-Reported Outcomes and Quality of Life Endpoints
SPIRIT	Standard Protocol Items: Recommendations for Interventional Trials
VASB	Visual Analogue Scale for Breathlessness survey
VHI	Voice Handicap Index
WOMAC	Western Ontario and McMaster Universities Osteoarthritis Index
